# Acute Graft Versus Host Disease After Kidney-Pancreas Transplant

**DOI:** 10.7759/cureus.40415

**Published:** 2023-06-14

**Authors:** James Lopez, Vishal Devarkonda, Moe Thuzar, Roberto Silva, Hugo Akabane

**Affiliations:** 1 Hematology and Medical Oncology, Louisiana State University Health Sciences Center, Shreveport, USA; 2 Internal Medicine, Louisiana State University Health Sciences Center, Shreveport, USA; 3 Pathology, Louisiana State University Health Sciences Center, Shreveport, USA

**Keywords:** unexplained shock, acute graft vs host disease, graft, solid organ transplant, transplant

## Abstract

Acute graft vs. host disease (aGVHD) results from newly transplanted donor immune cells recognizing recipient tissues as foreign, leading to end-organ damage. Diagnosing aGVHD typically involves a combination of clinical evaluation, histological examination, laboratory tests, and imaging studies. Although typically associated with allogeneic stem cells transplant and less frequently with liver or small bowel transplants, solid organ transplant GVHD (SOT-GVHD) associated with kidney-pancreas transplants is exceedingly rare. Our patient presented with pancytopenia unexplained by typical causes. He developed classical aGVHD findings of fever, diarrhea, rash, and abnormal liver tests. Our case underscores the importance of keeping a broad differential when evaluating solid organ transplant patients.

## Introduction

Acute graft-versus-host disease (aGVHD) frequently occurs as a complication following allogeneic hematopoietic stem cell transplantation, a treatment method employed for hematological malignancies. Incidence rates reported range from 9% to 50% among patients who undergo allogeneic HCT using a genetically matching human leukocyte antigen (HLA)-identical sibling as the donor [1]. Graft-versus-host disease (GVHD) can manifest as either an acute or chronic condition, where the primary characteristic involves the dysfunction of organs due to the detrimental effects of donor lymphocytes on the recipient's end-organ tissues [2].

The pathogenesis of acute graft-versus-host disease (aGVHD) is a complex process involving several interrelated factors, including activating donor T cells, producing pro-inflammatory cytokines, and recruiting immune cells to target tissues. The process begins with activating donor T cells, recognizing recipient tissues as foreign, and initiating an immune response. The T cells become activated by interactions with recipient antigen-presenting cells (APCs) and cytokines, stimulating their proliferation and differentiation into effector cells. The effector T cells then migrate to target tissues, where they cause tissue damage and inflammation through the release of pro-inflammatory cytokines, such as tumor necrosis factor-alpha (TNF-alpha), interleukin-1 (IL-1), and interleukin-6 (IL-6). These cytokines recruit additional immune cells, such as macrophages and neutrophils, to the target tissues, leading to further tissue damage and inflammation. The tissue damage caused by the effector T cells and other immune cells can result in various clinical manifestations, depending on the organs involved. For example, in the skin, tissue damage can lead to developing the characteristic rash of aGVHD [3]. At the same time, in the gastrointestinal tract, it can cause damage to the mucosal lining and result in diarrhea and abdominal pain [2]. While a small degree of donor-host immune response is desirable in allogeneic stem cell transplant (in the form of graft-versus-leukemia effect), it is only detrimental in solid organ transplant.

Graft-versus-host disease (GVHD) is a rare occurrence following liver transplantation (LT), with an incidence rate ranging from 0.5% to 2%. This condition is more commonly observed in patients who undergo small bowel transplantation, affecting approximately 5% of individuals [4]. The underlying pathogenesis is related to donor lymphocytes present in the transplanted organ. Common presenting signs and symptoms include a maculopapular or desquamating rash, fever, watery (bloody or non-bloody) diarrhea, transaminitis, or hyperbilirubinemia. Various grading systems can grade acute GVHD; common grading systems include the Glucksberg grade and the Mount Sinai Acute GVHD International Consortium (MAGIC) grading system [2]. Diagnosis can be confirmed by tissue biopsy demonstrating T-cell infiltration of the affected organ [5]. A limited number of treatment options are available for graft-versus-host disease (GVHD), typically involving immune suppression with corticosteroids to reduce the activity of donor lymphocytes and the use of anti-thymocyte globulin. However, it is essential to note that discontinuing immunosuppressive therapy can exacerbate GVHD symptoms and should be avoided. On the contrary, consideration should be given to increasing immunosuppressive treatment to manage the condition better [4,6].

## Case presentation

**Figure 1:**
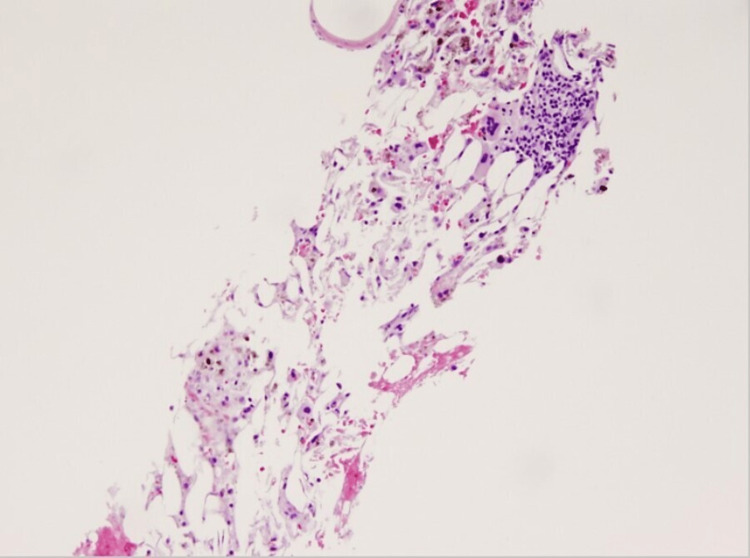
Bone marrow core showing low cellularity for age. Panhypoplasia and presence of dispersed erythropoietic elements and megakaryocytes. Scattered macrophages with hemosiderin are noticed.

A young adult male with a history of deceased donor kidney-pancreas transplant for type 1 diabetes mellitus complicated by end-stage renal disease was admitted to an outside hospital for unexplained pancytopenia and fevers for the past month. The patient only received blood transfusions with non-irradiated blood products before admission. He was on immunosuppression with prednisone, tacrolimus, and mycophenolate for his dual solid organ transplant, which occurred three months before his current presentation. He was on valganciclovir for cytomegalovirus (CMV) prophylaxis. The patient had no localizing symptoms of infection despite high fevers. An extensive infectious workup, including systemic imaging, opportunistic viral and fungal studies (CMV, BK virus, adenovirus, HHV-6, EBV, VZV, HSV, parvovirus), and blood and urine cultures, was negative. A bone marrow biopsy was performed to evaluate his worsening pancytopenia, which showed <10% cellularity (Figure 1), with a normal flow cytometry analysis, cytogenetics, and fluorescent in situ hybridization (FISH). Tacrolimus, mycophenolate, and valganciclovir were held with the presumptive diagnosis of drug-induced aplastic anemia; his prednisone dose was increased to compensate for stopping other immunosuppressive agents. He received two weeks of daptomycin, meropenem, and micafungin but had persistent fevers. For this reason, the patient was transferred to our institution for further evaluation and management.

Upon arrival at our institution, his physical exam was initially unremarkable (Table 1).

**Table 1:** Patient labs on arrival at our facility.

Test	Result	Reference Range
White blood cell count (WBC)	Low (0.04)	4.5-11.0 K/µL
Hemoglobin (Hgb)	Low (6.6)	13.5-17.5 g/dL
Platelet count	Low (32)	150-450 K/µL
Alkaline phosphatase	High (374)	40-150 U/L
Aspartate aminotransferase (AST)	High (97)	8-48 U/L
Alanine aminotransferase (ALT)	High (157)	7-55 U/L
Aspergillus antigen	Positive (3.071)	Negative
Beta-glucan	High (105)	Negative

**Figure 2:**
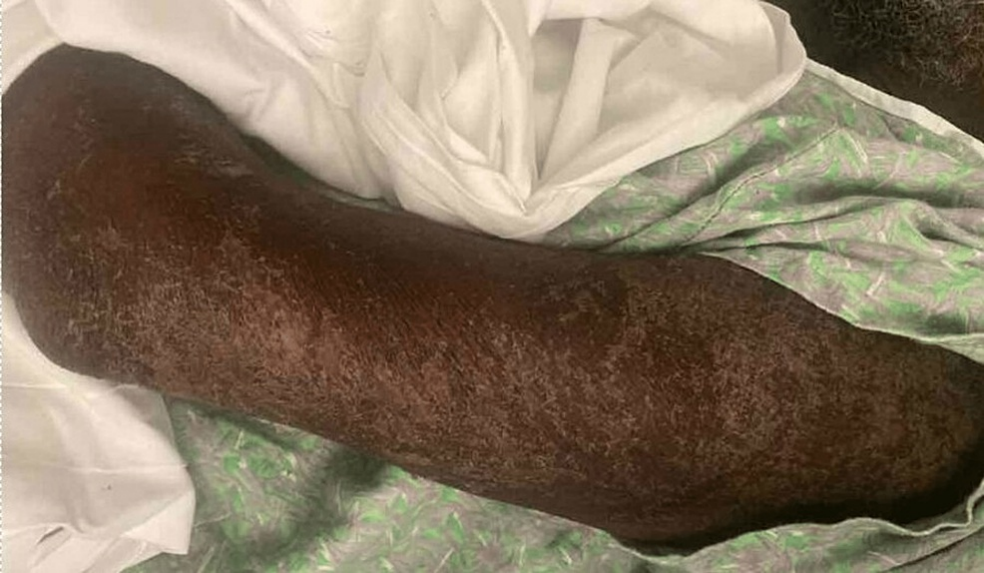
Example of patient’s rash from the posterior left arm.

Patient blood and urine cultures, as well as opportunistic viral infections, were repeated and resulted in negative. On day 3 of hospitalization, he developed profuse watery diarrhea. Stool studies, including ova and parasites, stool culture, and *Clostridium difficile* testing, were negative. A chest, abdomen, and pelvis computed tomography (CT) scan demonstrated multifocal pneumonia and diffuse colitis. A repeat bone marrow biopsy was attempted, but no aspiration could be obtained. Soon afterward, he developed a desquamating rash with depigmented skin underneath (Figure 2). The rash began on the face and spread to involve the torso and proximal arms and thighs. He also developed worsening transaminitis and direct hyperbilirubinemia throughout hospitalization; abdominal ultrasound was unremarkable, and the patient could not tolerate a magnetic resonance cholangiopancreatography (MRCP) scan.

The patient was transfused with irradiated and leuko-reduced blood products. He was started on vancomycin, meropenem, valacyclovir (for viral prophylaxis), and amphotericin B for neutropenic fever. For his pancytopenia, tacrolimus, mycophenolate, and valganciclovir were held due to their known significant myelosuppressive side effect profile. Despite broad-spectrum antimicrobial coverage, he continued to have persistently high fevers of 103°F or higher throughout hospitalization. Holding myelosuppressive therapy for three weeks did not improve his pancytopenia. Due to persistent pancytopenia, granulocyte-colony stimulating factor and eltrombopag were trialed without success.

**Figure 3:**
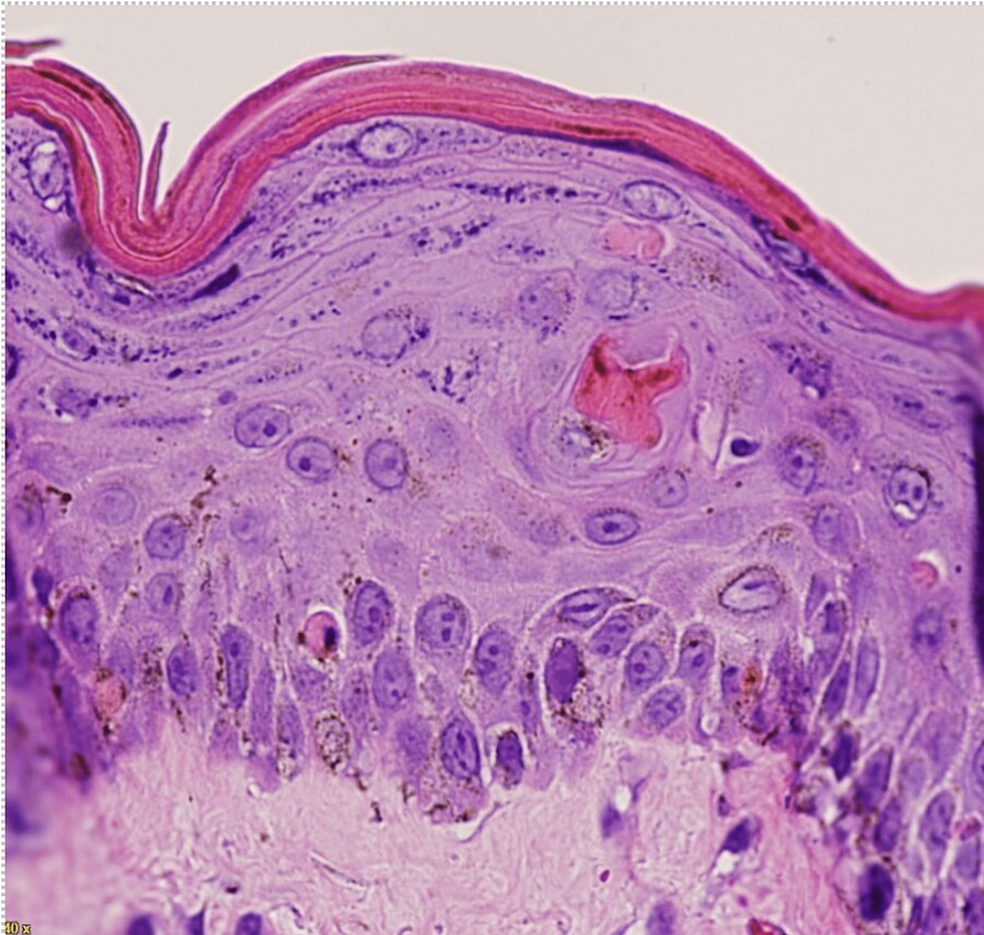
Skin biopsy showing mild hyperkeratosis, mild acanthosis, discrete spongiosis, and multiple necrotic keratinocytes dispersed on all layers of the epidermis.

The presence of colitis, desquamating rash, and a history of non-irradiated blood transfusions narrowed the differential diagnosis to acute GVHD related to blood transfusion versus kidney/pancreas transplant. The patient was started on oral budesonide and octreotide for GVHD-associated diarrhea and methylprednisolone 250 mg daily. A skin biopsy of an affected area of the skin showed evidence of T-lymphocyte invasion (Figure 3). The patient was started on horse-derived anti-thymocyte globulin (ATG) at 40 mg/kg daily for four days with tacrolimus. Despite these interventions, the patient’s clinical status declined, with worsening liver function and hypotension. Blood cultures resulted in multi-drug-resistant *Acinetobacter baumannii*. Unfortunately, the patient expired due to septic shock. Chimerism testing was sent on specimens from the patient and kidney donor, posthumously resulting in kidney-donor chimerism. Donor lymphocytes represented 94% of circulating T cells, confirming the diagnosis of kidney transplant-associated GVHD.

## Discussion

SOT-aGVHD is a rare but serious complication of solid organ transplantation. Mortality approaches 80% by five years from symptom onset, and the disease is often promptly fatal [4]. Many patients die from infections or refractory bone marrow failure [5]. Due to its rarity, SOT-GVHD is primarily reported in the medical literature as scarce case reports and small case series. Intestinal and liver transplantation have the highest risk of SOT-GVHD. Even rarer is SOT-GVHD after kidney-pancreas transplant, with only three other cases reported in the literature since 1997 [7].

Symptoms are similar to aGVHD, often seen in allogeneic stem cell transplants used to treat hematological malignancies [8]. aGVHD often injures three major target organs: the skin, gastrointestinal tract, and liver. The symptoms can be graded on the severity with various grading systems, most commonly the Glucksberg and MAGIC scoring systems. The pathophysiology of SOT-GVHD involves minor histocompatibility antigen mismatches between the donor T-cells and recipient tissue [2].

Diagnosis is often difficult due to disease rarity and other confounding factors. For example, chronic immunosuppressive and antimicrobial therapy often causes side effects similar to SOT-GVHD. As well, symptoms may not present concurrently, further inhibiting a prompt diagnosis. Diagnosis can be made with tissue biopsy demonstrating classical findings of aGVHD, as in this case, lymphocyte infiltration of the affected organ. Prior cases have used chimerism testing [5], routinely used in allogeneic stem cell transplant recipients to monitor allograft and liver transplant patients [9]. In our patient's case, a skin biopsy confirmed invasion by T cells, and chimerism testing proved the presence of donor lymphocytes. These results were crucial in confirming the diagnosis and could be used in future cases to guide treatment.

The successful response to treatment is crucially dependent on early diagnosis and prompt initiation of appropriate treatment. The first line of treatment is corticosteroids, in addition to immune suppression drugs such as tacrolimus. Due to the scarcity of SOT-GVHD, treatment options for the steroid-refractory disease are mainly derived from stem cell-associated aGVHD; there is no standard second-line therapy [9]. However, for glucocorticoid-resistant aGVHD, the recommended treatment is ruxolitinib, based on superior efficacy and modest toxicity shown in a phase 3 trial. Ruxolitinib is a selective inhibitor of Janus kinase (JAK) 1 and JAK2 and is effective in treating glucocorticoid refractory aGVHD. Other second-line treatments for glucocorticoid (GC)-resistant aGVHD include mycophenolate mofetil (MMF), etanercept, pentostatin, alpha-1 antitrypsin (AAT), sirolimus, and extracorporeal photopheresis (ECP). The choice of treatment beyond ruxolitinib varies among institutions and is influenced by availability, cost, and patient preference [10-13].

To better understand solid organ transplant graft-versus-host disease (SOT-GVHD) and improve patient outcomes, it is crucial to focus on better data acquisition and collaboration. SOT-GVHD is a rare condition that may be underreported, making it essential to establish stronger partnerships involving transplant registries and gather comprehensive data. Improved data acquisition would enhance awareness, facilitate a deeper understanding of the condition, and help develop standardized diagnostic criteria and treatment protocols. By investing in research and fostering collaborative efforts, we can advance the management of SOT-GVHD and improve patient care.

## Conclusions

In conclusion, this case report highlights the rare occurrence of solid organ transplant graft-versus-host disease (SOT-GVHD), specifically following a kidney-pancreas transplant. SOT-GVHD is a serious complication that can lead to significant morbidity and mortality. The clinical presentation of SOT-GVHD mimics that of acute GVHD seen in allogeneic stem cell transplantation involving the skin, gastrointestinal tract, and liver. Diagnosing SOT-GVHD can be challenging due to its rarity and similarity to other complications of solid organ transplantation. Tissue biopsy demonstrating lymphocyte infiltration and chimerism testing can aid in confirming the diagnosis. Treatment primarily relies on corticosteroids and immunosuppressive agents, with anti-thymocyte globulin being a potential second-line therapy. However, there is a lack of standardized treatment protocols due to the rarity of SOT-GVHD. Early recognition and initiation of appropriate treatment are crucial for improving patient outcomes. This case emphasizes the importance of considering SOT-GVHD in the differential diagnosis of solid organ transplant patients with unexplained clinical manifestations, and further research is needed to understand better and manage this rare condition.
